# Cultural collectivism, intimate partner violence, and women's mental health: An analysis of data from 151 countries

**DOI:** 10.3389/fsoc.2023.1125771

**Published:** 2023-03-30

**Authors:** Ravi Philip Rajkumar

**Affiliations:** Department of Psychiatry, Jawaharlal Institute of Postgraduate Medical Education and Research (JIPMER), Puducherry, India

**Keywords:** collectivism, intimate partner violence, depression, suicide, epidemiology

## Abstract

Culture, defined as the distinctive, learned beliefs and patterns of behavior that are particular to a given group or community, is a key determinant of mental health. The cultural dimension of individualism-collectivism, which measures the extent to which a given society accords importance to individuals as opposed to larger groups, has been associated with cross-national variations in mental health outcomes such as depression and suicide. However, this cultural dimension is also associated with variations in the frequency of intimate partner violence (IPV), which has a significant and sustained adverse impact on women's mental health. This study examines the relationships between individualism-collectivism, the frequency of IPV, and rates of depression and suicide in women, based on data from 151 countries. In this data set, IPV was significantly associated with age-standardized rates of depression and suicide in women, even after adjusting for demographic variables. Cultural collectivism was positively correlated with IPV, but this relationship was significantly influenced by national income and women's educational attainment. In multivariate analyses, IPV, but not cultural collectivism, remained significantly associated with depression in women. These results highlight the importance of screening for and addressing IPV in women seeking mental health care, particularly in low- and middle-income countries where cultural and economic factors may both increase the risk of IPV and delay or impede its reporting.

## Introduction

Culture has been defined by Hofstede as “the programming of the human mind by which one group of people distinguishes itself from another group.” In other words, culture refers to shared values, symbolic meanings and patterns of behavior that are specific to a given community, and which are acquired through learning from one's social environment (Hofstede et al., 2010). Because of its pervasive influence on all aspects of human life, culture is an important determinant of mental health (Campo-Arias et al., 2021). A proper understanding of the link between different aspects of culture, mental health outcomes, and the variables mediating this association is essential when planning and implementing mental health services at the community level (Alegria et al., 2022).

Several schemata for the classification of cultural values into distinct dimensions have been proposed by researchers (Hsu et al., 2013). Regardless of the specific classificatory system adopted, there is agreement among researchers in this field that cultures can be meaningfully described in terms of the importance accorded to the individual as opposed to that accorded to the wider group or community. This cultural dimension is referred to as *individualism-collectivism*. In an individualistic culture, which is typical of North American and European countries, there is a greater emphasis on individual liberty and a more “loosely-knit” social framework. In a collectivist culture, which is more typical of Asian and African countries, there is a more “tightly-knit” social framework and a subordination of individual desires and freedoms to the wellbeing of a larger social group (Hofstede et al., 2010; Wagner, 2022).

It is often stated that collectivism is associated with a reduced risk of depression and suicide; however, the underlying reality is more complex (Abramov and Peixoto, 2022). Initial studies of the link between cultural collectivism and depression did show a negative correlation between these two variables at the cross-national level. However, the authors of these studies also highlighted the need to consider the “goodness of fit” between genotype and social environment when interpreting these results (Chiao and Blizinsky, 2010; Way and Lieberman, 2010). Subsequent research in individual subjects has confirmed the need for a nuanced interpretation of the link between individualism-collectivism and mental health, with both individualism and collectivism linked to specific adverse outcomes (Kasof, 2009; Chu, 2015; Liu et al., 2017). Cultural collectivism may promote mental health through both through its effects on individual psychological processes, such as improved self-regulation and cognitive fluency (Li et al., 2018; Medina et al., 2019) and its social concomitants, such as enhanced social support in the face of adversity (Fu et al., 2007; Moscardino et al., 2010; Ariapooran et al., 2018).

However, every cultural adaptation represents a “trade-off” between advantages and disadvantages, and there are aspects of collectivism that can be harmful to an individual's mental health. Notions of family honor and shame, which are prevalent in collectivist cultures, can lead to a “culture of silence” surrounding traumatic situations such as sexual abuse (Haboush and Alyan, 2013), intimate partner violence (Ahmad et al., 2009), or violence within the family in general (Sawrikar, 2019). This leads to delayed or absent help-seeking and prolonged exposure to trauma. Such negative impacts may be specific to particular sub-groups within a population, such as women (Towns and Adams, 2009) and sexual minorities (Lowe et al., 2021), and are often passed over in discussions of the link between collectivism and mental health. There is also some evidence that in collectivistic societies, the impact of loneliness on physical and mental health may be greater than in individualistic cultures (Beller and Wagner, 2020). Finally, a special problem is posed by cultures in transition from a more collectivistic to a more individualistic set of values (Istiqlal et al., 2022). In these settings, the relationship between collectivism and mental health may be non-linear and difficult to predict (Berman et al., 2014). Adding to this complexity is the role played by economic factors such as poverty and income inequality, which are more common in collectivist cultures and are themselves risk factors for depression (Steptoe et al., 2007). It is clear that any evaluation of the link between individualism-collectivism and depression should attempt to account for both “positive” and “negative” confounding factors.

From a public health perspective, the relationship between individualism-collectivism, intimate partner violence (IPV) and women's mental health is of particular importance. Systematic reviews of the available evidence have found that exposure to IPV is associated with a nearly 2-fold increase in the risk of depression and suicide in women, even after adjusting for potential confounding factors (Devries et al., 2013; Bacchus et al., 2018). It is estimated that about 9–28% of depressive symptoms or disorders in women may be related to exposure to IPV (Beydoun et al., 2012). According to a recent World Health Organization Report, around 23–31% of women aged 15–49 who have ever been married or in a relationship have experienced IPV in their lifetime. Though no country or culture is free of IPV, there are marked variations across regions, ranging from a relative minimum of 10–15% in certain European countries to a maximum of over 40% in certain Asian, African and South American countries (World Health Organization, 2021a). This distribution approximates to a marked difference between individualistic and collectivistic societies; however, it is not possible to conclude from this that cultural collectivism is associated with higher rates of IPV *per se*. Nevertheless, an analysis of data from 52 countries found a significant positive association between IPV and more collectivistic cultural values (Archer, 2006).

Collectivistic societies are also associated with higher gender inequality and a lower social approval of divorce or separation (Pelham et al., 2022), both of which may lead to women remaining in abusive relationships. This relationship may be further moderated or mediated by individual demographic or environmental exposures. For example, a meta-analysis of factors associated with IPV across cultures, grouped using Hofstede's index of individualism-collectivism, found that age and relationship satisfaction were stronger predictors of IPV in individualistic countries, while witnessing parental IPV was a stronger predictor of IPV in collectivistic countries (Mallory et al., 2016). Likewise, a meta-analysis of research from a collectivist country found that education and family income were both negatively associated with the risk of IPV (Nikparvar et al., 2021).

The aim of the current study was to examine cross-sectional associations between cultural collectivism, intimate partner violence, and two specific mental health outcomes (depression and suicide) across countries, while correcting for possible confounding factors.

## Materials and methods

As data on IPV across countries was available only for a single time point, a cross-sectional ecological analysis was undertaken for the current study, keeping in mind the limitations associated with this approach. In this study, correlations between estimated cultural collectivism, lifetime risk of IPV, and the prevalence of depression as well as the suicide rate for women were examined across 115 countries. These analyses were adjusted for the possible confounding effects of women's education, per capita income, and a composite measure of gender inequality, as these factors were independently associated with a reduced risk of IPV in an earlier meta-analysis. Though there are certain limitations inherent in this form of cross-national analysis, they are valuable in identifying “macro”-level social, cultural and economic factors that can affect mental health in diverse populations (Meda et al., 2022).

### Data sources

Data on IPV was obtained from the World Health Organization's publication entitled “Violence against women prevalence estimates, 2018” (World Health Organization, 2021a). This monograph provides estimates of the lifetime prevalence of IPV in women for a total of 151 countries, provided as a percentage, based on data reported by United Nations member countries and multilevel statistical modeling. This process involved the collection of all representative published studies on the prevalence of IPV from WHO member countries, amounting to a total of 307 national and sub-national studies from 154 countries and regions (Maheu-Giroux et al., 2022).

The Global Collectivism Index (GCI) was used to estimate the position of each country on the individualism-collectivism continuum. This measure, published in 2022, is the first estimate of individualism-collectivism that covers the majority of the Earth's population, providing data on 188 countries and territories (Pelham et al., 2022). The GCI was constructed based on six indicators that have been found to predict individualism-collectivism: total fertility rate, living arrangements, stability of marriage, religiosity, collective transportation and in-group bias. Positive GCI scores indicate collectivistic cultural values, while negative scores indicate individualistic values.

Though the GCI was developed with the stated aim of providing a measure of individualism-collectivism that reduces Western cultural biases, it is partly based on a measure of motor vehicle sharing, which is inconsistent across low- and middle-income countries (Ingram and Liu, 1999). Therefore, as an independent measure of cultural individualism-collectivism, data on the Hofstede index of individualism-collectivism (HOF-IC) was retrieved from the Hofstede Institute's database (Hofstede, Insights, 2022). This measure is based on survey data from 115 countries, and has been used in studies examining the relationship between individualism-collectivism and mental health-related outcomes (Bailey and Kind, 2010; Gonda et al., 2011). The HOF-IC is scored from 0 to 100, with higher scores indicating cultural individualism and lower scores indicating cultural collectivism. Associations between the HOF-IC, IPV and mental health were not the primary outcome measures of this study, but were included as an additional measure of convergent validity.

Depression and suicide were selected mental health outcomes in this study due to their consistent association with IPV in earlier meta-analyses. The estimated prevalence of depression in women for each country was obtained *via* a database query from the Institute for Health Metrics and Evaluation (IHME), which provides estimates of the prevalence of depression by age and gender for each country and region based on Global Burden of Disease 2019 estimates (Global Burden of Disease Collaborative Network, 2020).

Suicide rates for women in each country were obtained from the World Health Organization's publication “Suicide worldwide in 2019: Global Health Estimates” (World Health Organization, 2021b), which provides crude and age-standardized estimates of the suicide rate per 100,000 population for 183 countries and territories. For 60 of these countries, data on suicides was obtained from direct reports and was considered to be of good quality. For the remaining 123 countries, which were largely low- and middle-income countries, statistical models were used to supplement available data, and estimated suicide rates were generated. To minimize the confounding effect of age on both the risk of IPV and mental health outcomes, age-standard estimates were used for both the prevalence of depression and the suicide rate in women.

Data on the educational status of women in each country was obtained from the United Nations' Human Development Report for the year 2019 (United Nations Development Programme, 2020), which provides data on average years of schooling for men and women for all member countries. From the same source, data was obtained on the Gender Inequality Index (GII), a composite measure of women's health, higher education, workforce participation and representation in government. The GII is scored from 0 to 1, with lower scores indicating higher equality and vice versa. Information on income, operationalized as the gross national income (GNI) per capita, was obtained for the year 2018 from the World Bank's database (The World Bank, 2022).

### Data analysis

All variables included in this study were tested for normality prior to further analysis. As none of the variables conformed to a Gaussian distribution, non-parametric correlations were used for bivariate analyses.

In the first stage of the data analysis, non-parametric correlations (Spearman's rho and Kendall's tau) were used to examine the associations between the GCI, the prevalence of IPV, and rates of depression and suicide in women. As each of these methods has certain advantages as well as limitations (Puth et al., 2015), both were used in the current study.

In the second stage, partial bivariate correlations were computed to examine (a) whether the associations between the GCI, the prevalence of IPV, and mental health outcomes were significant after adjusting for income, education and gender inequality, and (b) whether the association between IPV and mental health was moderated by the GCI, and (c) whether the association between the GCI and mental health was altered when IPV was taken into consideration. Both unadjusted and bivariate correlations were two-tailed, with the significance level set at *p* < 0.05. The strengths of the observed correlations were quantified based on Spearman's rho, using standard guidelines for psychology and the social sciences, as follows: 0.1–0.39, weak; 0.4–0.69, moderate; 0.7 and above, strong (Akoglu, 2018).

Following these steps, a multivariate linear regression analysis was carried out to examine whether any associations identified in the bivariate analyses remained significant, taking the prevalence of depression and the suicide rate in women as the dependent variables. Independent variables were selected for inclusion in the regression analyses if they were correlated with the outcomes at *p* < 0.1 or less. In view of the significant multicollinearity observed between certain predictor variables, the backward method of linear regression was adopted.

As a secondary measure, bivariate and partial correlations were repeated using the HOF-IC instead of the GCI, as described above, to examine whether the results obtained in each case would be similar in magnitude and direction.

## Results

Data from a total of 151 countries was included in the current study. Descriptive statistics for all study variables of interest are presented in Table 1.

**Table 1:** Descriptive statistics for all variables analyzed in the current study.

**Variable**	**Number of countries for which data was available**	**Shapiro-Wilk test statistic**	**Median (inter-quartile range)**	**Maximum**	**Minimum**
Intimate partner violence, lifetime prevalence (%)	151	0.943^*^	24.00 (13.50)	53.00 (Kiribati)	10.00 (Armenia)
Global collectivism index	143	0.966^*^	0.20 (1.29)	1.61 (Senegal)	−1.43 (Sweden)
Hofstede index of individualism-collectivism	101	0.907^*^	30.00 (32.00)	91 (United States)	6 (Guatemala)
Depression in women, age-standardized prevalence (%)	151	0.983	4.64 (1.58)	7.90 (Gambia)	2.45 (Colombia)
Female suicide rate, age-standardized (per 100,000 population)	145	0.715^*^	3.90 (3.70)	34.60 (Lesotho)	0.70 (Grenada)
Average years of education per adult woman	141	0.939^*^	8.90 (5.80)	13.50 (United States)	1.00 (Burkina Faso)
Gross national income per capita, standard $	123	0.840^*^	12398.10 (23486.50)	80522.20 (Luxembourg)	724.40 (Burundi)
Gender inequality index	134	0.950^*^	0.381 (0.334)	0.740 (Papua New Guinea)	0.037 (Switzerland)

^*^indicates a significant deviation from a Gaussian distribution (Shapiro-Wilk *p* < 0.05).

### Bivariate correlations between collectivism, IPV, and mental health outcomes

A complete correlation matrix for all unadjusted bivariate correlation analyses is presented in Table 2. It can be seen that the prevalence of IPV is positively correlated with both the prevalence of depression in women (ρ = 0.25, *p* = 0.002) and the female suicide rate (ρ = 0.27, *p* < 0.001), though the strength of these associations is modest. The GCI was positively correlated with the prevalence of depression in women (ρ = 0.20, *p* = 0.018), but not with the female suicide rate. A stronger, “moderate” correlation was observed between the GCI and the prevalence of IPV (ρ = 0.55, *p* < 0.001).

**Table 2:** Correlation matrix of unadjusted bivariate correlations between individualism-collectivism, intimate partner violence, mental health outcomes, and possible confounding factors.

**Variable**	**1 Dep-W**	**2 Suicide-W**	**3 IPV**	**4 GCI**	**5 Education**	**6 GNI**	**7 GII**
1	**-**	0.28 (<0.001)^**^0.20 (<0.001)^**^	0.25 (0.002)^**^0.20 (<0.001)^**^	0.20 (0.018)^*^0.12 (0.032)^*^	−0.33 (<0.001)^**^−0.21 (<0.001)^**^	−0.18 (0.049)^*^ −0.11 (0.069)	0.31 (<0.001)^**^ 0.20 (<0.001)^**^
2		-	0.27 (<0.001)^**^0.19 (0.001)^**^	0.05 (0.549)0.02 (0.701)	−0.07 (0.367) −0.05 (0.420)	−0.13 (0.150) −0.08 (0.220)	0.05 (0.534)0.02 (0.782)
3			-	0.55 (<0.001)^**^0.38 (<0.001)	−0.51 (<0.001)^**^−0.34 (<0.001)^**^	−0.56 (<0.001)^**^ −0.38 (<0.001)^**^	0.62 (<0.001)^**^0.43 (<0.001)^**^
4				-	−0.86 (<0.001)^**^−0.68 (<0.001)^**^	−0.91 (<0.001)^**^ −0.73 (<0.001)^**^	0.88 (<0.001)^**^0.70 (<0.001)^**^
5					-	0.86 (<0.001)^**^0.67 (<0.001)^**^	−0.84 (<0.001)^**^−0.65 (<0.001)^**^
**6**							−0.90 (<0.001)^**^−0.73 (<0.001)^**^

All correlations are given in the form: Spearman's rho (significance), Kendall's Tau-B (significance).

^*^Significant at *p* < 0.05; ^**^significant at *p* < 0.01.

Dep-W, estimated prevalence of depression in women (%); Suicide-W, estimated suicide rate per 100,000 women; IPV, lifetime prevalence of intimate partner violence (%); GCI, Global Collectivism Index; Education, average years of education per adult woman; GII, Gender Inequality Index; GNI, gross national income per capita ($).

When examining potential confounding variables, the level of education among adult women was negatively correlated with the prevalence of depression (ρ = −0.33, *p* < 0.001), but not with the suicide rate. GNI per capita was marginally correlated with the prevalence of depression, though there was inconsistency between the results obtained using Spearman's (*p* = 0.049) and Kendall's (*p* = 0.069) methods. IPV was negatively correlated with both women's education (ρ = −0.51, *p* < 0.001) and GNI per capita (ρ = −0.56, *p* < 0.001), and positively correlated with the GII (ρ = 0.62, *p* < 0.001). The GII was positively correlated with the prevalence of depression (ρ = 0.31, *p* < 0.001) but not the suicide rate. Cultural collectivism was strongly and negatively correlated with women's education, GNI and the GII to the point of multicollinearity (absolute value of ρ > 0.8 for all three correlations).

When using the HOF-IC as a measure of individualism-collectivism (Supplementary Table 1), this variable was negatively but non-significantly correlated with IPV (ρ = −0.13, *p* = 0.181) and positively and significantly correlated with both the prevalence of depression (ρ = 0.20, *p* = 0.044) and the suicide rate (ρ = 0.27, *p* = 0.007) in women, though the strength of these associations was weak. The differences in the directions of the observed correlations between the GCI and HOF-IC are due to the opposite scoring schemes used by each measure; higher GCI scores indicate a collectivistic cultural orientation, while higher HOF-IC scores indicate an individualistic orientation. There was a moderate-to-strong correlation between both measures of individualism-collectivism (ρ = 0.65, *p* < 0.001), though this did not reach the level of multicollinearity, indicating an acceptable level of convergence between these estimates of cultural orientation. The HOF-IC was positively correlated with women's education and GNI, but negatively correlated with the GII.

### Partial correlation analyses

In the first partial correlation analysis (Table 3), the associations between the GCI, the prevalence of IPV, and the frequency of depression and suicide among women were examined, controlling for GNI, years of education in women, and the GII. In these analyses, the associations between IPV and depression (ρ = 0.36, *p* < 0.001) and suicide (ρ = 0.30, *p* = 0.001) remained significant, and were slightly stronger than those observed in the unadjusted analyses. The adjusted associations between the GCI and both depression and suicide were not significant, though a weak negative association between the GCI and suicide was observed using Spearman's partial correlation (ρ = −0.23, *p* = 0.016). Similarly, the unadjusted positive correlation observed between the GCI and the prevalence of IPV was no longer significant when conditioned on income, education and gender inequality.

**Table 3:** Partial correlation analyses between individualism-collectivism, intimate partner violence, and mental health outcomes, controlled for confounding factors.

**Variable**	**1 GCI**	**2 IPV**	**3 Dep-W**	**4 Suicide-W**
1	-	−0.15 (0.103) −0.01 (0.862)	−0.03 (0.773)0.01 (0.899)	−0.23 (0.016)^*^ −0.09 (0.151)
2		**-**	0.36 (<0.001)^**^0.27 (<0.001)^**^	0.30 (0.001)^**^0.21 (0.001)^**^
3			-	0.34 (<0.001)^**^0.24 (<0.001)^**^

All analyses are conditioned on gross national income per capita, average years of education in women, and gender inequality index. All correlations are given in the form: Spearman's partial rho (significance), Kendall's partial Tau-B (significance).

^*^Significant at *p* < 0.05; ^**^significant at *p* < 0.01.

Dep-W, estimated prevalence of depression in women (%); Suicide-W, estimated suicide rate per 100,000 women; IPV, lifetime prevalence of intimate partner violence (%); GCI, Global Collectivism Index.

In the second partial correlation analyses, the association between IPV and both depression (ρ = 0.25, *p* = 0.002) and suicide (ρ = 0.30, *p* < 0.001) in women remained significant when conditioned on the GCI. On the other hand, the positive correlation between the GCI and the prevalence of depression in women was not significant after adjusting for the frequency of IPV (ρ = 0.02, *p* = 0.746).

In the third partial correlation analysis, the negative correlation between gross national income and IPV remained significant, albeit attenuated, when correcting for the GCI (ρ = −0.231, *p* = 0.011). On the other hand, the negative correlation between women's education and IPV was not significant after adjusting for the GCI (ρ = −0.10, *p* = 0.244). The correlation between the GII and IPV, though attenuated when conditioned on the GCI, also remained statistically significant (ρ = 0.37, *p* < 0.001).

Analyses of a similar nature using the HOF-IC revealed that, after adjusting for GNI, education and the GII, this measure was positively correlated with the prevalence of IPV (ρ = 0.28, *p* = 0.010) and depression (ρ = 0.34, *p* = 0.002), but not the suicide rate (Supplementary Table 1).

### Linear regression analyses

As only a single variable (IPV) was significantly associated with the suicide rate in women, linear regression could not be performed for this variable. The results of the multivariate linear regression analysis for the prevalence of depression in women is presented in Table 4. In this model, which attained statistical significance overall (*F* = 11.42, *p* < 0.001), three variables—the prevalence of IPV, the GII, and the average years of education for women—were retained as significant. IPV was positively associated with depression, while the GII and education were negatively associated with this variable. Variance inflation factors were below 4 for all variables, ruling out significant multicollinearity. Taken together, these three variables explained about 21% of the variance in the prevalence of depression in women (*R*^2^ = 0.239, adjusted *R*^2^ = 0.218).

**Table 4:** Multivariate linear regression analyses of the variables associated with depression in women.

**Variables entered in the model**	**Variable(s) excluded**	**Variable(s) included in the final model**	**Regression coefficient (β)**	**Significance level (*p*)**	**Variance inflation factor**
GCI	GNI	GII	−0.32	0.007^**^	1.92
IPV	GCI	IPV	0.41	<0.001^**^	1.37
GNI		Education	−0.36	0.001^**^	1.72
Education					
GII					

^**^Significant at *p* < 0.01.

IPV, lifetime prevalence of intimate partner violence (%); GCI, Global Collectivism Index; Education, average years of education per adult woman; GNI, gross national income per capita ($).

## Discussion

The current study was a preliminary attempt at elucidating the complex relationship between cultural individualism-collectivism, intimate partner violence, and adverse mental health outcomes in women at a cross-national level.

### Relationship between individualism-collectivism and mental health outcomes in women

In this study, higher scores on the GCI—which indicate more collectivistic cultural values—were positively correlated with the prevalence of depression, but not the suicide rate, in women. The strength of this correlation was weak (ρ <0.4), and it was no longer significant after adjusting for the effects of IPV, or after controlling for demographic variables (income and education) and gender inequality. In the linear regression analysis, the GCI was not significantly associated with depression. Taken together, these results suggest that a simple linear association between individualism-collectivism and depression in women is unlikely. After correcting for intimate partner violence and socioeconomic status, collectivism may have a modest protective effect against suicide in women, which is consistent with some of the earlier results of cross-national analyses (Chiao and Blizinsky, 2010; Way and Lieberman, 2010; Li et al., 2021) and of individual research in specific populations (Lake et al., 2022). This effect may reflect the protective value of social support through informal networks involving extended family or friends. Though such support is difficult to quantify, it is likely to exert a significant influence on the mental health of women who are victims of IPV. There is evidence that the reactions of one's social circle to the disclosure of IPV has a considerable effect on the mental health and wellbeing of victims. Such reactions are themselves influenced by the attitudes of those receiving such disclosures (Dworkin et al., 2019; Ullman, 2023), and these attitudes, whether positive or negative, are themselves shaped by local cultural values (Dery et al., 2022; Zark and Satyen, 2022). This suggests that attempts to either enhance these local support networks (Cao et al., 2021) or promote attitudinal change among community leaders or “gatekeepers” (Dery et al., 2022), may be helpful in preventing or mitigating the effects of IPV on women's mental health.

Qualitative research from a low-income country suggests that collectivistic values may be associated with a stronger “sense of order,” fostering hope and partially countering the effects of economic deprivation; however, such values may also contribute to a sense of entrapment which runs counter to hope (Eggerman and Panter-Brick, 2010) and may trigger depression as well as suicide attempts. It is also well-known that certain emotion regulation strategies, more commonly seen in women, may contribute to their increased risk of depression (Nolen-Hoeksema, 2012). Some authors have suggested that culture and gender may interact to influence the effectiveness of these strategies, leading to observable differences in the prevalence of depression across countries, but this hypothesis requires further testing (Vikan et al., 2009; Kwon et al., 2013; Ireland et al., 2015; De Vaus et al., 2018).

### Relationship between intimate partner violence and mental health outcomes in women

In contrast to the mixed findings observed for individualism-collectivism, IPV was consistently and significantly associated with both depression and suicide in women, even after adjusting for demographic confounders and gender inequality. This association is consistent with the results of several meta-analyses (Beydoun et al., 2012; Devries et al., 2013; Bacchus et al., 2018; Hawcroft et al., 2019; Insan et al., 2022; Vicard-Olagne et al., 2022), which reveal a consistent link between IPV and depression regardless of the definition of IPV, the type of study considered (cross-sectional or longitudinal), or the measure of depression examined (any depressive disorder, post-partum depression, depressive symptoms). IPV is a chronic, traumatic stressor that is recurrent and often escalating in severity, and this leads to a sense of entrapment and hopelessness that results in depression. The presence of depression in a victim of IPV may further increase vulnerability to further IPV, creating a vicious cycle that is often difficult to break (Mazza et al., 2021). The traumatic effects of IPV may persist for several years even after separation from an abusive spouse or partner, leading to chronic or recurrent depression (Ford-Gilboe et al., 2023).

In certain cases, particularly in collectivistic Asian cultures, women may present to mental health services with medically unexplained symptoms: such symptoms are not only indicative of undiagnosed depression, but may represent a metaphorical or symbolic means of communicating their experience of physical violence (Wong et al., 2016). Due to this, it is possible that the association between IPV and depression was underestimated in the current study.

Ongoing IPV also affects treatment outcomes for depression. For example, a recent meta-analysis of psychological interventions for female victims of IPV, focusing on low- and middle-income countries, found that these interventions were ineffective in bringing about a significant improvement in depression (Keynejad et al., 2020). Similar interventions in the same patient group, conducted in high-income countries with a more individualistic culture, were effective in alleviating depression (Hameed et al., 2020). These divergent findings suggest that a failure to protect women from IPV not only increases their risk of depression, but makes them less likely to benefit from otherwise effective forms of treatment. Therefore, the identification of IPV, and the availability of effective measures to protect women from further victimization, is essential if depression is to be effectively diagnosed and treated, particularly in women from collectivistic cultures (Connelly et al., 2013).

### Relationship between individualism-collectivism and intimate partner violence

In the current study, the relationship between individualism-collectivism and IPV was not significant after adjusting for demographic factors. However, the converse was also partially true; after adjusting for individualism-collectivism, IPV was only weakly correlated with national per capita income, and was not significantly correlated with women's education. Thus, while demographic factors may significantly mediate the association between cultural values and IPV, a direct contribution from certain aspects of culture cannot be entirely ruled out. Some of these, such as an emphasis on family honor and a reluctance to discuss intra-familial problems with “outsiders,” have been consistently reported in studies of migrant and ethnic minority women in Western cultures (Hulley et al., 2022) as well as in Asian and African settings (Mshweshwe, 2020; Arisukwu et al., 2021; Selim et al., 2022). Other values that have been reported to contribute to IPV in collectivist settings include notions of self-sacrifice associated with the female gender role (Natal, 2022), acceptance of cultural myths regarding the acceptability of violence among men (Toplu-Demirtas et al., 2022), and exaggerated notions of masculinity and patriarchy (Mshweshwe, 2020; Arisukwu et al., 2021). Though certain cultural models do incorporate a dimension of masculinity-femininity (Hofstede et al., 2010), this dimension is more a measure of assertiveness / competitiveness vs. consensus / cooperation, and may not map exactly onto beliefs regarding gender roles. Further research into cultural sexual scripting and masculine roles in relation to collectivism and IPV is warranted (Fleming et al., 2015; Willie et al., 2018).

### The contribution of income, women's education, and gender inequality

The correlation between income and IPV observed in this study was stronger than that observed between collectivism and IPV. While an association between national wealth and the national prevalence of IPV has been identified by earlier researchers, other economic factors such as income inequality and unemployment may also contribute to variations in IPV across countries (Kebede et al., 2022). Financial hardship may contribute to the perpetration of IPV by increasing levels of childhood adversity, chronic stress, mental disorder and alcohol use in men (Thompson and Kingree, 2004; Papadakaki et al., 2009), and may also act as a barrier to women seeking help, particularly when they lack economic independence (Goodman et al., 2009). As the association between income and IPV was substantially weakened after correcting for individualism-collectivism, it is unlikely that cross-national variations in IPV can be ascribed solely to economic factors. The average level of education attained by adult women was negatively associated with both IPV and depression in women, which may reflect the association between this variable and the general level of gender equality and women's empowerment in a given society (Gomez et al., 2011); moreover, education may be protective against IPV in its own right (Zhao et al., 2022).

In this study, a composite measure of gender inequality, covering inequities in health, higher education, employment and political participation, was positively correlated with both depression and intimate partner violence in bivariate analyses; this is consistent with existing literature showing a link between gender inequality and depression in women (Van de Velde et al., 2021; Kim et al., 2023). However, this association was not replicated in the multivariate analysis, which is perhaps a reflection of the limitations inherent in such composite measures.

### Differences between two measures of collectivism

It is also important to note the effect of the choice of a measure of individualism-collectivism on the associations that were observed. Though there was a reasonable degree of agreement between the GCI and the HOF-IC (|ρ| = 0.70), important differences emerged in the analyses based on which index was considered. IPV was more strongly correlated with the GCI than with the HOF-IC; the HOF-IC, but not the GCI, was significantly associated with the suicide rate in women; and the HOF-IC remained significantly correlated with the prevalence of IPV and depression in women even after controlling for demographic factors and gender inequality. These divergences highlight the limitations inherent in using a single numerical index to quantify collectivism, particularly when these indices are computed at different levels of analysis. Thus, the HOF-IC is based on survey data from individuals across various countries, which is then extrapolated to the national level; the IPV, on the other hand, is based on six macro-level demographic variables measured directly at the national level. Moreover, many researchers distinguish between horizontal and vertical dimensions of both individualism and collectivism in their work (Germani et al., 2021; Young et al., 2021), and these dimensions may have distinct influences on factors such as subjective wellbeing and feelings of guilt, which in turn may influence outcomes such as depression and suicide. It is likely that further work on more sensitive and multi-dimensional measures of individualism-collectivism may clarify the relationship between this aspect of culture and outcomes such as IPV and mental illness.

### Strengths and limitations

This study, though preliminary in nature, is the first to examine the associations between collectivism, IPV and women's mental health across a large number of countries, including low- and middle-income countries. The use of partial and regression analyses permits a more accurate delineation of the links between these variables.

Nevertheless, certain limitations of the study must be borne in mind. First, due to limitations in longitudinal data availability for IPV, only cross-sectional analyses were performed, precluding any definitive conclusions regarding the causal effects of this variable on women's mental health. Such effects may be inferred from the agreement between the current study's results and those involving individual subjects, but cannot be confirmed. Second, the possibility of under-reporting of both IPV and mental health outcomes may have led to an underestimation of the association between these variables. Third. other variables which could influence the links between IPV and depression, such as alcohol consumption, unemployment, or measures of gender inequality, were not analyzed as due to a lack of large-scale cross-national data on these factors was not available for most of the countries studied. Fourth, as the analyses were based on nation-level data, they cannot be directly applied to individuals. Fifth, other mental health outcomes of interest, such as post-traumatic stress disorder, could not be assessed as they were not estimated in the Global Burden of Disease studies. Sixth, this study did not examine the role of potential protective factors, such as social support, cultural values that support the protection and empowerment of women, or the effectiveness of legislation against IPV at the community level (Whitaker, 2014). Seventh, there are certain common risk factors for both IPV and suicide, such as age, employment status and prior trauma exposure, that could not be analyzed as confounders in the current study due to a lack of cross-national data (Iovine-Wong et al., 2019). Finally, and perhaps most importantly, data on the outcome variables for this study—depression and suicide—was based on direct measurements only in a minority of countries; most estimates of these variables had to be derived through statistical modeling and extrapolation from available data, which was often inadequate or absent (Brhlikova et al., 2011; World Health Organization, 2021b). This problem was of less concern in the case of IPV, where a larger body of original research was available across countries (World Health Organization, 2021a). In addition, even when official data or published research was available, there is a tendency toward under-reporting of sensitive matters such as IPV and suicide (Tollefsen et al., 2012; Barbier et al., 2022). In the case of IPV, this may be due to barriers in reporting IPV-related offenses (Dandona et al., 2022); in the case of suicide, this may be because of legislation or culture-based stigmatization (Arya et al., 2020). Due to these factors, it is likely that the current study may have underestimated the strength of the association between IPV and mental health outcomes. Though this is a significant limitation, it also highlights the need for locally-based, confidential and woman-friendly processes for the reporting of IPV and related phenomena, both for the sake of their protection and for the collection of reliable data that can be used to guide policy and advocacy (Madden et al., 2019; Vatnar et al., 2021).

## Conclusion

The results of the current study, though provisional and subject to certain limitations, highlight the links between a specific dimension of culture and IPV; the consistency of the association between IPV and both depression and suicide in women; and the possible moderating effects of income, education and gender inequality on these associations in women. These results confirm and extend the work of earlier researchers, and highlight the need for culturally sensitive assessment of IPV in women presenting with depression, particularly in low-and middle-income countries. These results also suggest that structural change at multiple levels, including general economic development, measures aimed at promoting women's equality and empowerment, and the implementation of effective legal measures for the protection of women from IPV, is required to reduce the considerable burden of mental disorder associated with this form of violence.

## Data availability statement

The raw data supporting the conclusions of this article will be made available by the authors, without undue reservation.

## Author contributions

The author confirms being the sole contributor of this work and has approved it for publication.
