# Supplementary material for: Cultural collectivism, intimate partner violence, and women's mental health: An analysis of data from 151 countries
Source: Front Sociol. 2023 Mar 30;8:1125771. doi: 10.3389/fsoc.2023.1125771 (PMC10098113; doi:10.3389/fsoc.2023.1125771)
Supplement: Supplementary file 1 [file Table_1.DOCX]

**Supplementary Table 1: Unadjusted and adjusted correlations between Hofstede’s index of individualism-collectivism, intimate partner violence, and depression and suicide in women**

| **Variable** | **Intimate partner violence, lifetime prevalence (%)** | **Depression in women, age-adjusted prevalence (%)** | **Suicide rate in women, age-standardized** |
| --- | --- | --- | --- |
| **Hofstede’s index of individualism-collectivism, unadjusted** | -.13 (.181)  -.09 (.215) | .20 (.044)^*^  .15 (.033)^*^ | .27 (.007)^**^  .18 (.008)^**^ |
| **Hofstede’s index of individualism-collectivism, adjusted†** | .28 (.010)^*^  .12 (.119) | .34 (.002)^**^  .19 (.013)^*^ | .20 (.077)  .12 (.100) |

† Adjusted for gross national income, average years of schooling for women, and Gender Inequality Index

All correlations are given as Spearman’s rho (significance level) / Kendall’s Tau-B (significance level)

* Significant at *p* < .05

** Significant at *p* < .01
